# Resource utilization and cost saving analysis of subcutaneous versus intravenous trastuzumab in early breast cancer patients

**DOI:** 10.18632/oncotarget.18527

**Published:** 2017-06-16

**Authors:** Alberto Farolfi, Paolo Silimbani, Davide Gallegati, Elisabetta Petracci, Alessio Schirone, Mattia Altini, Carla Masini

**Affiliations:** ^1^ Department of Medical Oncology, Istituto Scientifico Romagnolo per lo Studio e la Cura dei Tumori (IRST) IRCCS, Meldola, Italy; ^2^ Oncology Pharmacy Unit, Istituto Scientifico Romagnolo per lo Studio e la Cura dei Tumori (IRST) IRCCS, Meldola, Italy; ^3^ Financial and Management Control, Istituto Scientifico Romagnolo per lo Studio e la Cura dei Tumori (IRST) IRCCS, Meldola, Italy; ^4^ Unit of Biostatistics and Clinical Trials, Istituto Scientifico Romagnolo per lo Studio e la Cura dei Tumori (IRST) IRCCS, Meldola, Italy; ^5^ Healthcare Administration, Istituto Scientifico Romagnolo per lo Studio e la Cura dei Tumori (IRST) IRCCS, Meldola, Italy

**Keywords:** breast cancer, economic evaluation, oncology, subcutaneous trastuzumab

## Abstract

We conducted an economic evaluation of intravenous (IV) *vs* subcutaneous (SC) trastuzumab for the treatment of patients with early breast cancer (EBC). Data of patients receiving adjuvant IV trastuzumab at our institute in 2014 were used to study three different treatment scenarios: 1) IV trastuzumab, 2) SC trastuzumab, and 3) IV trastuzumab during chemotherapy followed by SC trastuzumab. Our cohort included 114 patients with a median weight of 63.75 kg. Scenario 2 was the most time-saving treatment, with 71.7% reduction in preparation time and 89.3% reduction in chair time compared to scenario 1. Considering full costs, the mean costs per patient/year were € 14,233 ± 8,698 for scenario 1, € 14,272 ± 8,312 for scenario 2, and € 14,535 ± 8,646 for scenario 3 (*p* = 0.959). When mean body weight was > 65.2 kg, the mean cost was lower in scenario 2 than in scenario 1. Scenario 2 proved a valuable time-saving and cost-saving option. A shift from IV to SC trastuzumab should be considered, especially in capacity-constrained oncology departments.

## INTRODUCTION

Humanized monoclonal antibody trastuzumab has been shown to have a significant survival benefit for patients with early breast cancer (EBC) [1]. Until a few years ago, trastuzumab was available only as an intravenous (IV) infusion, whose dose was calculated according to the patient's body weight. The loading dose was administered for at least 90 minutes followed by a maintenance dose of at least 30 minutes [2]. The subcutaneous (SC) formulation administered via hand-held syringe contained a fixed dose of 600 mg trastuzumab and the excipient recombinant human hyaluronidase (rHuPH20). It has sought approval from the European Medicines Agency for EBC as an alternative to the conventional IV trastuzumab, based on comparable pharmacokinetics and efficacy results, and similar safety profiles provided by a non-inferiority study [3]. SC trastuzumab has proved superior over IV according to patient preference, because it saved time (administered for 5 minutes without any need of a loading dose), causing less pain, discomfort and side-effects [4]. SC administration results in a faster and equally safe method of treatment delivery, enabling physicians and nurses to save time and increase the number of patients that can be treated during their working hours [5].

Nevertheless, both formulations must be adequately prepared before administration. Manual preparation of cytotoxic drugs has always been considered a high-risk procedure, given the prolonged exposure to carcinogens during handling [6] and the high level of therapy personalization, which leads to greater chances of error [7].

The introduction of robots in the drug preparation process has aimed to reduce both the number of errors and the risk for the staff from exposure to carcinogenic agents, improving product quality in terms of drug dosage accuracy and sterility. In our previous study on the quality and economic implications of manual *vs* automated preparation of antineoplastic drugs, including trastuzumab, we demonstrated that both procedures were accurate. The automated preparation yielded better quality maintenance standards (*i.e.* higher accuracy of the active ingredient concentration) and lower risk for operators [8]. Manual dosing may lead to occasional errors which are difficult to track down: we found that manual preparation resulted in > 10% discrepancy between prescribed and prepared dose of trastuzumab [8].

The results of the HannaH and PrefHer studies demonstrated that SC trastuzumab is an efficacious, well-tolerated treatment, and a valuable option for patients and healthcare professionals. However, concerns about increasing costs of the fixed dose of SC trastuzumab are limiting its use. Basing our investigation on the Italian Health System key principles of equitable and sustainable care, we evaluated the resource utilization associated with the administration of SC trastuzumab compared with IV trastuzumab in patients with HER2-positive EBC. Advanced settings were not considered, as an IV trastuzumab biosimilar [9, 10], whose direct costs are still unknown, will soon be available.

We analyzed direct and indirect costs associated with both formulations in the adjuvant setting in 3 scenarios in order to determine the most performing in terms of cost saving: IV trastuzumab (scenario 1), SC trastuzumab (scenario 2), and IV trastuzumab during chemotherapy followed by SC trastuzumab after chemotherapy (scenario 3). Scenario 3 was designed considering that the PrefHer trial showed that the safety profile was not affected in any way by the switch from SC to IV or viceversa [11].

## RESULTS

### Direct costs

In 2014, 114 breast cancer patients with a median weight of 63.75 kg (range 55–74.9) were treated with adjuvant IV trastuzumab. Table 1 shows the main patient characteristics. Considering the adverse events, 4 patients (3.5%) prematurely interrupted the treatment because of cardiotoxicity (asymptomatic left ventricular ejection fraction drop). Normal heart function was restored once treatment had been discontinued without sequelae. Other five patients (4.38%) experienced a significant reduction in left ventricular ejection fraction, without the need of treatment interruption. Five patients (4.38%) had relapsed by the time of the analyses.

**Table 1:** Patient characteristics

	No.	(%)
Median age, years [range]	56	[33–82]
Median weight, kg [range]	63.75	[42–95]
Histology		
Ductal	110	(96.5)
Lobular	2	(1.75)
Other	2	(1.75)
Nuclear grade		
G1	0	-
G2	28	(24.6)
G3	86	(75.4)
ER status		
≥ 1%	80	(70.2)
< 1%	34	(29.8)
PgR status		
≥ 1%	63	(55.3)
< 1%	51	(44.7)
Ki67		
High (≥ 20%)	16	(14)
Low (< 20%)	98	(86)
Tumor stage		
T1	64	(56.2)
T2	42	(36.8)
T3	5	(4.4)
T4	3	(2.6)
Nodal stage		
N0	69	(60.5)
N1	32	(28.1)
N2	8	(7)
N3	5	(4.4)
Type of chemotherapy		
Adjuvant	96	(84.2)
Neoadjuvant	18	(15.8)
Chemotherapy scheme		
AC or EC	5	(4.4)
AC or EC followed by wP	64	(56.2)
FEC followed by wP or D	21	(18.4)
wP	11	(9.7)
DC	3	(2.6)
TCH	3	(2.6)
CMF	4	(3.5)
Vinorelbine	3	(2.6)

Abbreviations: AC, adriamycin and cyclophosphamide; CMF, cyclophosphamide, methotrexate and fluorouracil; EC, epirubicin and cyclophosphamide; D, docetaxel; DC, docetaxel and cyclophosphamide; ER, estrogen receptor; FEC, fluorouracil epirubicin and cyclophosphamide; PgR, progesterone receptor; TCH, docetaxel, carboplatin and trastuzumab; wP, weekly paclitaxel.

IV trastuzumab was delivered in 1,292 cycles, corresponding to 937 cycles of SC trastuzumab in scenario 2, and 543 cycles of IV trastuzumab, and 749 cycles of SC trastuzumab in scenario 3. Overall, 372,214 mg IV trastuzumab were administered, corresponding to 562,200 mg SC trastuzumab in scenario 2, with a difference of 189,986 mg in drug dosage (Δ = 51.0% in trastuzumab dose) (Table 2). The total cost of the drugs were as follows: € 1,544,688 for scenario 1, € 1,613,514 for scenario 2, and € 1,612,275 for scenario 3, for a total of 77,710 mg IV trastuzumab delivered in 543 cycles and 449,400 mg SC trastuzumab in 749 cycles. Given an average of 0.11% drug waste of trastuzumab during preparation, regardless of the days in which most costly therapies are prepared, the cost of drug waste amounted to € 1,699 and € 344 for scenarios 1 and 3, respectively. Consequently, the mean direct costs per patient were: € 13,655 ± 8,412, € 14,154 ± 8,243, and € 14,146 ± 8,514 for scenario 1, 2 and 3, respectively. Differences in direct costs among the three scenarios were not statistically significant (*p* = 0.832).

**Table 2:** Weight quartiles of patient population and trastuzumab in the two formulation doses infused per quartile

Weight quartiles	IV trastuzumab dose (mg)	SC trastuzumab dose (mg)	Δ (mg)
Q1 = 55 kg	78,138	154,800	76,662
Q2 = 63.7 kg	82,938	133,800	50,862
Q3 = 74.9 kg	107,514	152,400	44,886
Q4 = 95 kg	103,624	121,200	17,576
Total	372,214	562,200	189,986

Q, quartile; IV, intravenous; SC, subcutaneous

### Indirect costs

SC trastuzumab required an average preparation time 24.1% shorter than IV trastuzumab per cycle. Given the lower number of preparations in scenario 2, the overall preparation time was 71.7% shorter for SC trastuzumab. Mean preparation time per patient was 10.5 ± 6.3 hours, 3.0 ± 1.7 hours, and 3.7 ± 5.2 hours for scenarios 1, 2 and 3, respectively. This difference was statistically significant, p_omnibus_< 0.001 (p for scenario 2 *vs* 1 < 0.001, p for scenario 3 *vs* 1 < 0.001, and p for scenario 3 *vs* 2 < 0.001) (Table 3).

**Table 3:** Time per preparation and administration of trastuzumab in the three scenarios

	Scenario 1	Scenario 2	Δ (%)	Scenario 3	Δ (%)
Unit preparation time (sec)	844	641	–203 (24.1)	-	-
Overall preparation time (h)	120	34	–86 (71.7)	78	–42 (35)
Mean preparation time per patient (h)	10.5 ± 6.3	3.0 ± 1.7	–7.2 (71.7)	6.8 ± 5.2	–3.7 (35)
Unit administration time (min)					
Loading dose [No. of cycles]	90 [85]	5 [85]	–85 (94.4)	90 [53]*; 5 [32]	0
Maintenance dose [No. of cycles]	30 [1, 207]	5 [1, 212]	–25 (83.3)	30 [490]; 5 [717]	
Overall administration time (h)	731	78	–653 (89.3)	-387	–344 (47)
Mean administration time per patient (h)	6.4 ± 3.7	0.7 ± 0.4	–5.7 (89.3)	3.4 ± 3.2	–3.0 (47)

sec, seconds; h, hours; min, minutes

*The number of loading doses is lower than in scenario 1 because 32 patients received trastuzumab for the first time after chemotherapy and were thus directly “transferred” to subcutaneous trastuzumab.

Administration of SC trastuzumab in scenario 2 reduced nursing and chair time by an average of 89.3% compared with IV trastuzumab. In scenario 3, the overall time of administration was reduced by 47% (Table 3). Mean administration time per patient was 6.4 ± 3.7 hours, 0.7 ± 0.4 hours and 3 ± 3.2 hours for scenarios 1, 2 and 3, respectively. Again, these differences were statistically significant, p_omnibus_< 0.001 (p for scenario 2 *vs* 1 < 0.001, p for scenario 3 *vs* 1 < 0.001, and p for scenario 3 *vs* 2 < 0.001).

The preparation of one cycle of IV trastuzumab cost € 8.17, whereas one cycle of SC trastuzumab € 6.99. Mean preparation costs per patient were € 92.60 ± 55.80, € 57.50 ± 33.50, and € 84.80 ± 52.70. This led to a statistically significant difference among the three scenarios, p_omnibus_< 0.001considering scenario 2 *vs* scenario 1 (€ -35.14, *p* < 0.001), scenario 3 *vs* scenario 2 (€ -27.39, *p* < 0.001). This was not the case between scenarios 3 and 1 (€ -7.75, *p* = 0.681).

Occupational costs of outpatient clinic were different across scenarios: € 65,644 for scenario 1, € 7,012 for scenario 2, and € 34,753 for scenario 3 (Table 4). Mean outpatient clinic costs per patient were: € 575.82 ± 329.15, € 61.51 ± 35.82 and € 304.78 ± 284.06 for scenarios 1, 2 and 3 respectively, p_omnibus_< 0.001 (p for scenario 2 *vs* 1 < 0.001, p for scenario 3 *vs* 1 < 0.001, and p for scenario 3 *vs* 2 < 0.001).

**Table 4:** Mean cost per patient among the three scenarios

	Scenario 1	Scenario 2	Scenario 3	p_omnibus_
Direct cost	13,655 ± 8,412	14,154 ± 8,243	14,146 ± 8,514	0.832
Preparation cost	92.6 ± 55.8	57.5 ± 33.5	84.8 ± 52.7	< 0.001
Day hospital costs	575.82 ± 329.15	61.51 ± 35.82	304.78 ± 284.06	< 0.001
Global cost	14,233.2 ± 8,698.41	14,272.6 ± 8,312.63	14,535.3 ± 8,646.76	0.959

### Cost per patient

Considering the direct and indirect costs between scenarios, we calculated an overall cost of € 1,899,217 for scenario 1, € 1,902,374 for scenario 2, and € 1,933,681 for scenario 3, which equates to a mean cost per patient of € 14,233.20 ± 8,698.41 for scenario 1, € 14,272.60 ± 8,312.63 for scenario 2, and € 14,535.30 ± 8,646.76 for scenario 3. Mean global costs per patient among the three scenarios were not statistically significant (*p* = 0.959).

**Figure 1:**
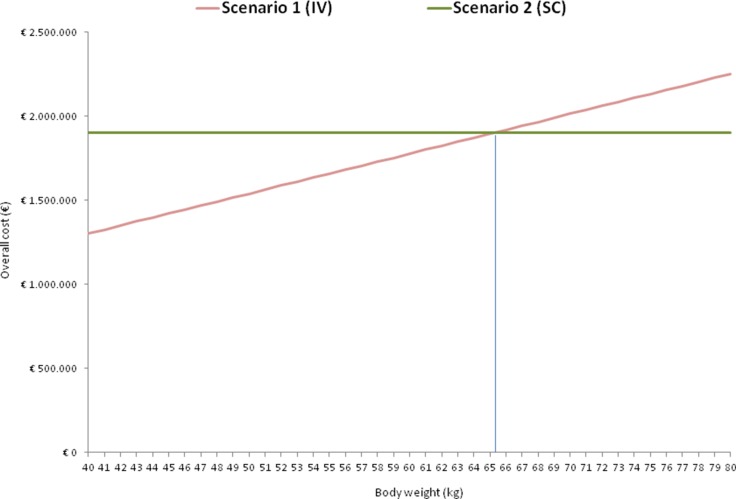
Overall cost per scenario according to body weight in scenarios 1 and 2

We subsequently designed a model considering all the costs associated with trastuzumab taking weight as a variable, only for scenarios 1 and 2, since scenario 3 proved non-performing for economical evaluation. The model in Figure 1 shows that if the mean weight of patients treated with adjuvant trastuzumab is > 65.2 kg (break even point), scenario 2 will be less expensive than scenario 1.

## DISCUSSION

SC trastuzumab proved to be non-inferior to IV trastuzumab in terms of event-free survival and overall survival in a large randomized trial with a median follow-up of over 40 months, with no difference in the frequency and/or severity of adverse events despite the different dosages of the formulations [12]. Considering the patients’ preference of SC over IV trastuzumab [4] and the saving of preparation and administration times, the SC formulation should be considered as a valuable clinical opportunity to improve the efficiency of HER2-positive breast cancer treatment delivery.

Our analysis was conducted to evaluate the economic implications of this new formulation with an innovative approach, which considered not only direct drug costs but also indirect costs. This led to the hypothesis of a switch in drug formulation from IV to SC. To this aim, we built three scenarios for the adjuvant treatment of HER2-positive EBC: 1) standard of care with the IV formulation; 2) SC trastuzumab only; 3) IV trastuzumab during chemotherapy followed by SC trastuzumab, in accordance with the PreHer trial, which showed no difference in safety for the prescribed sequence [11].

Even if mean global costs per patient were not statistically different, scenario 3 was shown to be the worst in terms of cost saving with a mean cost per patient/year of € 14,535. This result was against our expectations, as the indirect costs were much higher than in scenario 2. Scenario 2 was slightly more expensive than scenario 1 in drug cost per patient (*i.e.* € 39.40 per patient/year), yet less expensive in terms of cost of time (*i.e.* opportunity cost): staff and other resources could be directed to other activities, *i.e.* patient management and treatment delivery. In addition, scenario 2 resulted more advantageous in terms of “value” (*i.e.* health and quality outcome for money spent), with benefits of patient time saving. Compared to scenario 1, the higher overall cost of scenario 2 lowered with the increase in the average patient body weight, resulting equal in cost (break even point) when mean body weight was > 65.2 kg (Figure 1).

As a previous study [8] demonstrated that the average preparation unit cost in terms of labor and waste was lower in automated than in manual procedures, we hypothesized a totally robotic drug preparation. Differences in resource utilization could therefore be less marked where manual preparation is still a standard of care.

In our study population, median body weight was 63.75 kg, whereas in the PrefHer trial was 68 kg and 66 kg in arms A and B, respectively [13]. Given that in the HannaH trial no difference in adverse events or pharmacokinetics was reported with respect to body weight [3], our evaluation supports even more the rationale for the choice of SC over IV trastuzumab, not to mention patient preference. The higher direct costs of SC trastuzumab are counterbalanced by the reduced resource utilization related to the time saved in drug preparation and administration.

These results are in line with the economical evaluation of a similar context in New Zealand: the switch between formulations reduced both the time spent in the clinic and the healthcare professional resources and consumables needed for administration, contributing to an overall reduction in healthcare costs [14]. As in the PrefHer trial, SC trastuzumab showed to save more patient chair and healthcare professional time than IV infusion [15].

Healthcare decision makers should consider that a change in the trastuzumab formulation from IV to SC will lead to a considerable, immediate rise in drug cost, yet counterbalanced by other economic advantages, such as the possibility to treat a greater number of patients, improving hospital accessibility, reducing waiting lists while maintaining standards of care, and increasing treatment capacity by relieving capacity-constrained oncology departments. Hospitals with acceptable and manageable waiting lists and on lower budgets may find the use of SC trastuzumab inconveniently costly. Patient time-saving benefits from SC trastuzumab preparation and administration should also be taken into account.

This study has a few limitations that must be highlighted. First, the results of this study may not be generalized due to the different direct and indirect drug costs across countries. It can be hypothesized that scenario 3 will be the worst scenario, regardless of the actual costs. Patient preference, well-being, and quality of life were not assessed in this study, although discussed in the PrefHer study [4, 13]. By far the most common reason for patients to prefer SC trastuzumab was that it saved time [13], as our study shows. Moreover, the impact and the costs of indwelling IV catheter insertion, maintenance, and complication-related management were not included in the analysis. Finally, although we designed three different scenarios starting from a real-life situation (our 2014 experience), the scenarios might detach from the actual situation, in particular with respect to the number of patients treated and their mean body weight. To reach more generalizable findings, we reported the mean cost per patient/year and created a model with body weight as a variable.

## MATERIALS AND METHODS

In order to compare the resource utilization of the two trastuzumab formulations, we designed a simple model including all the patients treated with adjuvant trastuzumab whose data were retrieved from the institutional medical record database (Log80) from January 1st, 2014 to December 31st, 2014. The database collects all the pharmacy and medical data of the patients treated at the Istituto Scientifico Romagnolo per lo Studio e la Cura dei Tumori (IRST) IRCCS (Meldola, Italy). On the basis of this retrospective cohort study, we simulated that the same cohort would be treated with either IV or SC trastuzumab for the whole time or with IV trastuzumab during chemotherapy followed by SC trastuzumab.

Based on a previous work [8], we hypothesized that both formulations would be robotically prepared.

### Robotic drug preparation

The robot (ApotecaCHEMO^®^) is located in a closed, microbiologically controlled environment. Its anthropomorphic arm mechanically replicates the actions of a human operator. The system allows for continuous checks on the entire preparation process. All of the production steps, as well as incoming and outgoing materials, are checked and recorded by technological devices, such as sensors, photocells, a vision system, and barcode readers. Automatic identification of drugs, weight-checking system, and barcode labeling guarantee process traceability. Preparation time was calculated as the time between the first and last weighing of components.

### Resource utilization analysis

For each hypothesized model, we calculated: a) the direct cost of the drug used (only one type of trastuzumab is commercially available in Italy – Herceptin^®^ 150 mg, Roche): € 4.15/mg for IV formulation and € 2.87/mg for SC formulation; b) the differential gap (Δ) of milligrams of drug administered between the two different formulations; c) drug preparation time and unit costs for a healthcare professional; and d) drug administration time and unit costs for outpatient clinic management per hour. As patients may have received 2 mg/kg IV trastuzumab weekly during chemotherapy, 3 preparations were converted into one of SC trastuzumab administered every 3 weeks, maintaining preparation and administration times separate.

Costs for indwelling venous lines (*e.g*. central venous catheter – Portacath and Groshong) were not considered as patients had already had them fixed for previous chemotherapy. Unit costs for healthcare professionals were retrieved from the Italian National Contract, and were as follows: € 21/hour for a technician and € 60/hour for a hospital pharmacist. Unit costs for outpatient clinic management was € 89.80/hour retrieved from the personnel costs, resource utilization (excluding drug costs) and management cost for the total number of hours of outpatient clinic use.

For both formulations, the average time for preparation did not include the time for the transfer of supplies from the storage room to the pharmacy, the pre-labeling of the input material and the time for dressing change of the operators. The average preparation time was therefore calculated from the actual start time of reconstitution of drug vials and preparation of bags, labeling and identification of the preparation by means of a barcode scan.

### Statistical analyses

Descriptive statistics are reported as frequencies and percentages for categorical variables and as mean and standard deviation (SD) or median and range for continuous variables. The analysis of variance (ANOVA) was performed to compare the means among the three scenarios. Post-hoc comparisons were performed when the omnibus F-test was statistically significant. The Bonferroni method was used to adjust for multiple comparisons. A two-sided *p*-value < 0.05 was deemed statistically significant for all the analyses. All statistical analyses were carried out using STATA 10.1 statistical software (StataCorp, College Station, TX, USA).

## CONCLUSIONS

Data from our study demonstrated that treatment with SC trastuzumab should be initiated immediately, without switching from another treatment. SC trastuzumab proved a time-saving and a potential cost-saving treatment without significantly increasing the mean global costs per patient. This resulted in a reduction in resource utilization and an improvement of the capacity of oncology departments to treat a higher number of patients. We showed that economic evaluations of drugs should not only include the direct costs, but also the consequences of the different treatment modalities, *i.e.* indirect costs.
